# Misdiagnosis of Lyme Disease With Unnecessary Antimicrobial Treatment Characterizes Patients Referred to an Academic Infectious Diseases Clinic

**DOI:** 10.1093/ofid/ofz299

**Published:** 2019-07-05

**Authors:** Takaaki Kobayashi, Yvonne Higgins, Roger Samuels, Aurasch Moaven, Abanti Sanyal, Gayane Yenokyan, Paul M Lantos, Michael T Melia, Paul G Auwaerter

**Affiliations:** 1 Infectious Disease, University of Iowa Hospitals and Clinics, Iowa City, Iowa; 2 Sherrilyn and Ken Fisher Center for Environmental Infectious Diseases, Johns Hopkins University School of Medicine, Baltimore, Maryland; 3 Division of Pulmonary and Critical Care Medicine, Johns Hopkins University School of Medicine, Baltimore, Maryland; 4 University of Maryland School of Medicine, Baltimore, Maryland; 5 Johns Hopkins Biostatistics Center, Johns Hopkins Bloomberg School of Public Health, Baltimore, Maryland; 6 Medicine and Pediatrics, Duke University School of Medicine, Durham, North Carolina; 7 Infectious Disease, Johns Hopkins University School of Medicine, Baltimore, Maryland

**Keywords:** *Borrelia burgdorferi*, chronic Lyme disease, Lyme disease, tick-borne coinfections

## Abstract

**Background:**

Although Lyme disease is the most common vector-borne infection in the United States, diagnostic accuracy within community settings is not well characterized.

**Methods:**

A retrospective observational cohort study of patients referred to an academic center with a presumed diagnosis or concern for Lyme disease between 2000 and 2013 was performed to analyze diagnoses and treatments. Characteristics of those with Lyme disease and those misdiagnosed as having Lyme disease were compared.

**Results:**

Of 1261 patients, 911 (72.2%) did not have Lyme disease, 184 (14.6%) had active or recent Lyme disease, 150 (11.9%) had a remote history of Lyme disease, and 16 (1.3%) were identified as having possible Lyme disease. Patients without current Lyme disease were more likely to be female (odds ratio [OR], 1.56; 95% confidence interval [CI], 1.08–2.45), to have had symptoms for >3 months (OR, 8.78; 95% CI, 5.87–13.1), to have higher symptom counts (OR per additional symptom, 1.08; 95% CI, 1.02–1.13), to have had more Lyme-related laboratory testing (OR per additional laboratory test, 1.17; 95% CI, 1.03–1.32), and to have been diagnosed with what were regarded as coinfections (OR, 3.13; 95% CI, 1.14–8.57). Of the 911 patients without Lyme disease, 764 (83.9%) had received antimicrobials to treat Lyme disease or their coinfections. The percentage of patients established to have Lyme disease was lower than in earlier studies of referred populations.

**Conclusions:**

Among patients referred to an academic Infectious Diseases practice for Lyme disease, incorrect diagnoses and unnecessary antibiotic treatment were common, both for Lyme disease and for coinfections.

## Teaser

Of 1261 patients referred for evaluation of presumptive Lyme disease, only 26.5% were diagnosedaccurately. Those without current Lyme disease were more likely to be female, have a longer duration of symptoms, and be inaccurately diagnosed with co-infections.

Lyme disease is a spirochetal infection caused by *Borrelia burgdorferi* sensu lato transmitted to humans by the bite of infected *Ixodes* ticks. Lyme disease is the most common vector-borne infection in the United States, geographically spreading in recent decades [1, 2]. Although most patients respond to antibiotic therapy, some may experience persistent subjective symptoms such as fatigue and pain 6 months or longer after initial treatment. Among 8 US published studies, a median (range) of 11.5% (0%–40.8%) of cases were noted to have ongoing subjective symptoms [3]. The mechanisms for these lingering symptoms are not well understood, though multiple prospective, randomized, placebo-controlled trials have not found substantial or durable responses to additional antibiotic therapy, arguing against ongoing infection as an explanation [4, 5]. Post-treatment Lyme disease syndrome (PTLDS) criteria have been proposed to provide a framework for research but have not yet been clinically validated [6].

Although the term “chronic Lyme disease” (CLD) does not have a consensus definition, it is applied by some practitioners for patients with long-standing issues, such as fatigue, pain, and behavioral or neurocognitive difficulties, regardless of whether there is objective evidence of infection with *B. burgdorferi* [7]. Earlier studies suggested that Lyme disease is often overdiagnosed, leading to unnecessary antibiotic treatment [8–11]. However, the characteristics of those with a misdiagnosis of *B. burgdorferi* infection, including the CLD population, are not well known. This study evaluated the demographic characteristics, clinical history, laboratory test results, and antibiotic treatment history of patients referred to an academic infectious diseases clinical practice in Maryland for a Lyme disease consultation. The findings provide data on the accuracy of the clinical diagnosis and provide insights into community practices.

## METHODS

### Study Design and Patient Sample

This retrospective observational study was performed in a single center, an outpatient suburban infectious diseases clinic of the Johns Hopkins University School of Medicine located in Lutherville, Maryland. Patient charts were screened for a presumptive diagnosis of Lyme disease and for referral to rule out Lyme disease between January 1, 2000, and December 31, 2013. Persons younger than 12 years old were excluded, as the clinic did not treat children below this age. The anonymized data set was compiled by 4 medical research assistants (T.K., Y.H., R.S., A.M.) and subsequently reviewed independently by 2 infectious diseases physicians (M.M., P.A.) for accuracy of information and diagnoses. A standardized list of symptoms, physical examination findings, and laboratory data was used for clinical data extraction for every patient record. This study was approved by the Institutional Review Board of Johns Hopkins University School of Medicine.

Data on other infections previously diagnosed or treated in conjunction with Lyme disease were tabulated. These infections included other *Ixodes*-transmitted infections (*Anaplasma phagocytophilum*, *Babesia microti*), infections transmitted by other tick species (*Ehrlichia* spp., Rocky Mountain spotted fever, *Babesia duncani*), other infections not transmitted by ticks (*Bartonella*, mycoplasma, Epstein-Barr virus, parvovirus, dengue, malaria, and other parasites), and problems characterized as due to an infection (FL 1952 parasite, natural killer cell deficiency, hemobartonella). Non-tick-borne infections were included if they were believed to occur contemporaneously with the patient’s Lyme disease.

Results of *B. burgdorferi* antibody testing were used for clinical analysis only if performed using Food and Drug Adminstration–approved serologic methods; results of molecular testing were also used if performed by commercial or reference laboratories [12]. Results from nonstandard, laboratory-developed tests were collected but were not used for diagnosis at the referral clinic.

Patients were divided into 4 groups depending on symptoms, duration, objective findings, and Lyme disease diagnostic testing: (1) patients without Lyme disease, (2) patients with active or recent Lyme disease including PTLDS, (3) patients with remote Lyme disease, and (4) patients with possible Lyme disease. Patients without Lyme disease had no clinical findings or laboratory evidence of Lyme disease. Patients with active/recent Lyme disease had Lyme disease when evaluated at our facility or convincing evidence of infection within 2 years of evaluation. Patients with remote Lyme disease had symptoms that had started at least 2 years after complete recovery from an earlier episode of Lyme disease. This conservative time frame of a 2-year symptom-free interval was selected to effectively eliminate concern that the current symptoms were linked to a previous episode of Lyme disease. Patients with possible Lyme disease refers to patients who had unusual symptoms or examination findings with positive *B. burgdorferi* serology, but with clinical equivocation expressed as to whether the *B. burgdorferi* infection was causal.

Clinical and serological criteria for diagnosing Lyme disease were based on established criteria [6, 13]. Patients who were diagnosed with active/recent or remote Lyme disease were separated into groups of early Lyme disease if the patient experienced <3 months of symptoms before Lyme disease diagnosis (excluding arthritis), late Lyme disease if the patient experienced >3 months of symptoms before the Lyme disease diagnosis (excluding arthritis), Lyme arthritis, and PTLDS. If Lyme disease was diagnosed from the consultation but record review was insufficient to categorize temporally, then the patient received an unknown Lyme diagnosis. Clinical characteristics among the groups were compared. In addition, patients without Lyme disease and patients with a remote history of Lyme disease were combined into a category called patients without current Lyme disease. The patients without current Lyme disease were then compared with the active/recent Lyme disease patients to evaluate variables potentially useful for predicting when Lyme disease would not account for their consultation complaints. Lyme disease diagnostic rates in this study were also compared with previously published studies.

### Statistics

Data were expressed as counts and percentages for categorical variables and as means and standard deviations for continuous variables. For ordinal variables, medians with 25th and 75th percentiles or ranges are presented. To evaluate relationships between patient characteristics and Lyme disease status, the chi-square test or Fisher exact test was used for categorical variables. Analysis of variance or a nonparametric Kruskal-Wallis test was performed to assess group differences for continuous/ordinal variables. When analyzing differences in continuous and ordinal variables between dichotomized groups, *t* tests and nonparametric Mann-Whitney tests were used, respectively. Univariate and multivariable logistic regression models were used to identify predictors of patients without current Lyme disease and to estimate odds ratios. Patients with possible Lyme disease were excluded from the logistic regression analysis due to low numbers. Statistically significant variables in the univariate analyses were included in the multivariable logistic regression model. All hypothesis tests were performed at a 5% level of statistical significance. All analyses were conducted using the STATA 14 statistical software program.

## RESULTS

### Baseline Characteristics

Of 2854 new ID consultations, 1261 patients were referred or self-referred for a presumptive diagnosis of, or concern for, Lyme disease. The mean age of the 1261 patients was 45.7 years, and 779 (61.8%) were women. One patient (0.08%) was asymptomatic at presentation, seeking an explanation for a laboratory test result. Among the 1260 (99.92%) symptomatic patients, the median duration of complaints was 558 days, ranging from 1 day to 51 years. The 5 most commonly identified symptoms were arthralgia (71.3%), fatigue/malaise (66.8%), headache (42.1%), myalgia (40.8%), and sleep disturbance (34.3%). The 5 most common abnormal physical findings were rash other than erythema migrans (6.6%), joint swelling (5.9%), tender points (3%), objective sensory abnormality (2.1%), and motor weakness (1.5%). Although 139 (11%) coinfections were diagnosed before evaluation at the infectious diseases clinic, none of these infections were confirmed or treated based upon the evaluations performed in this study. Of these 139 putative coinfections, 61 (44%) were said to be caused by *Babesia microti* or *B. duncani*, 40 (29%) by Epstein-Barr virus, 30 (22%) by Bartonella, 11 (8%) by *Ehrlichia* spp., and 32 (23%) were attributed to other infectious agents.

The median antimicrobial treatment duration (25th–75th percentile) in the study population was 40 (21–84) days. The 5 most common antibiotics previously taken were doxycycline (908 patients, 72%), ceftriaxone (240 patients, 19%), amoxicillin (171 patients, 13.6%), cefuroxime axetil (107 patients, 8.5%), and azithromycin (105 patients, 8.3%). Receipt of more than 1 previous anti-infective course was reported by 473 patients (37.5%). Three or more laboratory tests for Lyme disease were noted in 659 patients (52.3%; 2-tier serologic testing was regarded as a single test if supplemental immunoblots were performed as a reflex to a positive or equivocal first-tier enzyme immunoassay [EIA]).

### Lyme Disease Status

**Figure 1.**
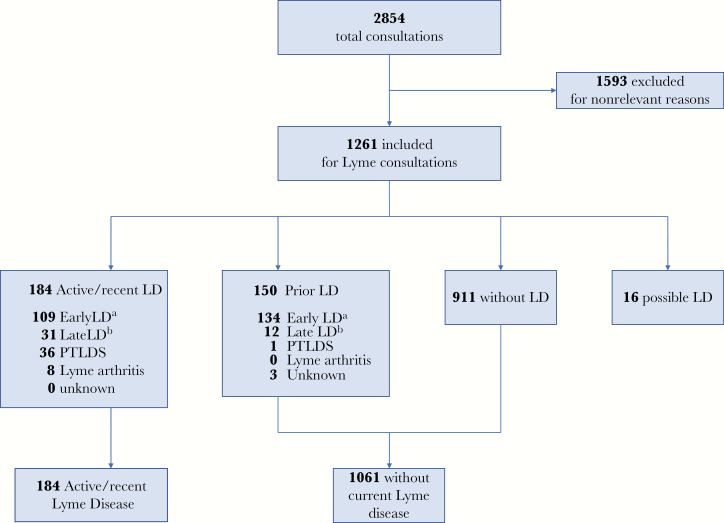
Study flowchart in the patient sale (n = 1261). ^a^Symptom duration <3 months before diagnosis of Lyme disease (excluding arthritis). ^b^Symptom duration >3 months before diagnosis of Lyme disease (excluding arthritis). Abbreviations: ALD, active/recent Lyme disease; LD, Lyme disease; PTLDS, consistent with post-treatment Lyme disease syndrome.

Patient demographic and medical history characteristics were compared across the 4 Lyme disease diagnostic groups (Table 1). The diagnostic determinations of the 1261 patients are presented in Figure 1: 911 (72.2%) patients did not have Lyme disease, 184 (14.6%) had active/recent Lyme disease, of whom 36 (2.9%) had PTLDS, 150 (11.9%) had remote Lyme disease, and 16 (1.3%) had possible Lyme disease.

**Table 1.** Baseline Demographic and Clinical History Characteristics in the Patient Sample (n = 1261)

	Total (n = 1261)	Patients w/o LD (n = 911)	Active/Recent LD (n = 184)	Remote LD (n = 150)	Possible LD (n = 16)	*P* Value
Age, mean (SD), y	45.7 (15.4)	45.13 (14.9)	44.75 (16.6)	48.69 (15.9)	61.19 (15.2)	.023
Female, No. (%)	779 (61.80)	608 (66.70)	89 (48.40)	77 (51.30)	5 (31.30)	<.001
Race, No. (%)						.290
White	1153 (91.40)	831 (91.20)	166 (90.20)	141 (94.00)	15 (93.80)	
African American	35 (2.80)	31 (3.40)	3 (1.60)	1 (0.70)	0 (0.00)	
Other	22 (1.70)	14 (1.50)	5 (2.70)	3 (2.00)	0 (0.00)	
Unknown	51 (4.00)	35 (3.80)	10 (5.40)	5 (3.30)	1 (6.30)	
Symptom duration^a^						<.001
0–3 mo, No. (%)	174 (13.80)	72 (7.90)	90 (48.90)	10 (6.70)	2 (12.50)	
3–6 mo, No. (%)	125 (9.90)	71 (7.80)	36 (19.60)	14 (9.30)	4 (25.00)	
>6 mo, No. (%)	962 (76.30)	768 (84.30)	58 (31.50)	126 (84.00)	10 (62.50)	
Median (p25–p75), d	558 (190–1483)	757 (270–1663)	95 (30–210)	580 (283–1513)	476 (152–957)	
Mean (range), d	1248 (1–18 518)	1466 (2–18 518)	261 (1–5356)	1178 (19–8060)	827 (17–4263)	
Symptoms, No. (%)						
Arthralgia	899 (71.30)	675 (74.10)	117 (63.60)	97 (64.70)	10 (62.50)	.002
Fatigue/malaise	842 (66.80)	634 (69.60)	113 (61.40)	86 (58.00)	8 (50.00)	.004
Headache	531 (42.10)	396 (43.50)	77 (41.80)	54 (36.00)	4 (25.00)	.227
Myalgia	514 (40.80)	388 (42.60)	62 (33.70)	61 (40.70)	3 (18.80)	.081
Sleep disturbance	433 (34.30)	351 (38.50)	31 (16.80)	49 (32.70)	2 (12.50)	<.001
Physical examination, No. (%)						
Other rash (not EM)	83 (6.60)	62 (6.80)	12 (6.50)	9 (6.00)	0 (0.00)	.932
Joint swelling	74 (5.90)	46 (5.00)	22 (12.00)	4 (2.70)	2 (12.50)	.001
Tender points	38 (3.00)	34 (3.70)	0 (0.00)	4 (2.70)	0 (0.00)	.008
Sensory abnormality	27 (2.10)	20 (2.20)	1 (0.50)	6 (4.00)	0 (0.00)	.102
Motor weakness	19 (1.50)	17 (1.90)	0 (0.00)	2 (1.30)	0 (0.00)	.171
History of coinfection, No. (%)	139 (11.00)	120 (13.20)	6 (3.30)	13 (8.70)	0 (0.00)	<.001
EIA,^b^ No. (%)						<.001
Positive	474 (46.5)	252 (33.7)	121 (87.1)	90 (75)	11 (78.6)	
Negative	494 (48.4)	455 (60.9)	14 (10.1)	24 (20)	1 (7.1)	
Equivocal	52 (5.1)	40 (5.4)	4 (2.9)	6 (5)	2 (14.3)	
Immunoblot,^c^ No. (%)						<.001
-IgM -IgG	256 (29.7)	225 (38.5)	10 (6.9)	19 (16.1)	2 (13.3)	
-IgM +IgG	105 (12.2)	50 (8.5)	29 (20)	20 (16.9)	6 (40)	
+IgM -IgG	364 (42.2)	271 (46.3)	48 (33.1)	43 (36.4)	2 (13.3)	
+IgM +IgG	138 (16)	39 (6.7)	58 (40)	36 (30.5)	5 (33.3)	
Anti-infective medication(s): duration						.003
None, No. (%)	165 (13.10)	147 (16.10)	12 (6.50)	2 (1.30)	4 (25.00)	
1–30 d, No. (%)	409 (32.40)	269 (29.50)	88 (47.80)	47 (31.30)	5 (31.30)	
31–90 d, No. (%)	413 (32.8)	272 (29.90)	65 (35.30)	70 (46.70)	6 (37.50)	
91–182 d, No. (%)	136 (10.80)	99 (10.90)	15 (8.20)	22 (14.70)	0 (0.00)	
>183 d, No. (%)	124 (9.80)	112 (12.30)	3 (1.60)	8 (5.30)	1 (6.30)	
Unknown, No. (%)	14 (1.10)	12 (1.30)	1 (0.50)	1 (0.70)	0 (0.00)	
Median (p25–p75), d	40 (21–84)	42 (21–90)	30 (21–51)	49 (28–81)	26 (10–47)	<.001
Mean (range), d	82 (0–2555)	90 (0–1440)	43 (0–240)	88 (0–2555)	38 (0–210)	
Total No. of anti-infectives prescribed (%)						.872
0	165 (13.10)	147 (16.10)	12 (6.50)	2 (1.30)	4 (25.00)	
1	623 (49.40)	412 (45.20)	103 (56.00)	99 (66.00)	9 (56.30)	
2	252 (20.00)	172 (18.90)	48 (26.10)	30 (20.00)	2 (12.50)	
3	111 (8.80)	83 (9.10)	17 (9.20)	10 (6.70)	1 (6.30)	
4	51 (4.00)	46 (5.00)	1 (0.50)	4 (2.70)	0 (0.00)	
5 or more	59 (4.70)	51 (5.60)	3 (1.60)	5 (3.30)	0 (0.00)	
Anti-infective meds, No. (%)						
Doxycycline	908 (72.00)	622 (68.30)	157 (85.30)	121 (80.70)	8 (50.00)	<.001
Ceftriaxone	240 (19.00)	167 (18.30)	33 (17.90)	36 (24.00)	4 (25.00)	.242
Amoxicillin	171 (13.60)	109 (12.00)	34 (18.50)	26 (17.30)	2 (12.50)	.022
Cefuroxime	107 (8.50)	85 (9.30)	13 (7.10)	9 (6.00)	0 (0.00)	.292
Azithromycin	105 (8.30)	93 (10.20)	3 (1.60)	8 (5.30)	1 (6.30)	<.001
Total No. of tests per individual						<.001
None, No. (%)	32 (2.50)	20 (2.20)	10 (5.40)	2 (1.30)	0 (0.00)	
1 or 2, No. (%)	570 (45.20)	381 (41.80)	118 (64.10)	68 (45.30)	3 (18.80)	
3 or more, No. (%)	659 (52.30)	510 (56.00)	56 (30.40)	80 (53.30)	13 (81.30)	
Median	3	3	2	3	3	

Abbreviations: EIA, enzyme immunoassay; EM, erythema migrans; LD, Lyme disease.

^a^One asymptomatic person excluded from the Past LD group for purposes of calculating duration.

^b^Sample size for EIA test results was 1020, instead of 1261, due to missing data.

^c^Sample size for immunoblot results was 863, due to missing or incomplete data, or not performed as EIA screen was negative.

Patients without current Lyme disease were more likely to be female (66.7%; *P* < .001) compared with those with Lyme disease. Symptom duration was shortest in the acute/recent Lyme group (median, 93 days; *P* < .001), although patients without Lyme disease had a median symptom duration of 557 days. The most common symptom was arthralgia in all 4 groups. Arthralgia was reported more frequently in patients without Lyme disease (74.1%; *P* < .002), as were fatigue (69.6%; *P* < .004) and sleep disturbance (38.5%; *P* < .001). Joint swelling was identified most often in patients with acute/recent Lyme (12%; *P* < .001). In patients without Lyme disease, 83.9% received antibiotics before referral, whereas 93.5% of patients with active/recent Lyme disease had received antibiotic therapy before evaluation.

### Comparison Between Patients Without Current Lyme Disease and Those With Active/Recent Lyme Disease

Patients without Lyme disease and patients with remote Lyme disease were combined into a category called patients without current Lyme disease and compared with active/recent Lyme disease subjects, as shown in Table 2. There were no statistically significant differences between the 2 groups’ mean age (*P* = .471) or race (*P* = .327). Female gender was more prevalent in those without current Lyme disease (64.6% vs 48.4%; *P* < .001). Patients without current Lyme disease had longer symptom duration than did those with active/recent Lyme disease (median number of days, 733 vs 95; *P* < .001). Among the 5 most common symptoms, arthralgia (72.8% vs 63.6%; *P* = .011), myalgia (42.3% vs 33.7%; *P* = .028), and sleep disturbance (37.7% vs 16.8%; *P* < .001) were more common in those without current Lyme disease. A history of tick-borne coinfections or other infections diagnosed as accounting for symptoms was reported more frequently in patients without current Lyme disease (12.5% vs 3.3%; *P* < .001). Abnormal physical examination findings were notable for more joint swelling in patients with acute/recent Lyme disease (12.0% vs 4.7%; *P* < .001), whereas tender points were described more frequently in patients without current Lyme disease (3.6% vs 0%; *P* = .004). The rate of EIA positivity was higher in those with active/recent Lyme disease (87.1% vs 39.4%; *P* < .001). On average, patients without current Lyme disease took anti-infective medications for a longer duration (42 days vs 30 days; *P* = .009) and had more Lyme disease diagnostic tests performed (median tests per individual, 3 vs 2; *P* < .001). In the 594 patients without current Lyme disease who had a symptom duration >30 days, antibiotic use was notably higher in patients with a positive IgM immunoblot (293/313, 93.6%, vs 233/281, 82.9%, with a negative IgM immunoblot; *P* < .001). Patients without current Lyme disease reported more frequent use of antidepressant medications (24.4% vs 10.3%; *P* < .001).

**Table 2.** Comparison Between Patients With and Without Recent/Active Lyme Disease^a^

	Without Current LD (Patients Without LD and Patients With Remote LD) (n = 1061)	Acute/Recent LD (n = 184)	*P* Value
Age, mean (SD), y	45.6 (15.1)	44.7 (16.6)	.471
Female gender, No. (%)	685 (64.60)	89 (48.0)	<.001
Symptom duration			<.001
<3 mo, No. (%)	82 (7.7)	90 (48.90)	
3–6 mo, No. (%)	85 (8.00)	36 (19.60)	
>6 mo, No. (%)	894 (84.30)	58 (31.50)	
Median (p25–p75)	733 (270–1650)	95 (30–210)	
Mean (range)	1426 (2–18 518)	261 (1–5356)	
Symptoms, No. (%)			
Arthralgia	772 (72.80)	117 (63.60)	.011
Fatigue/malaise	721 (68.00)	113 (61.40)	.082
Headache	450 (42.40)	77 (41.80)	.886
Myalgia	449 (42.30)	62 (33.70)	.028
Sleep disturbance	400 (37.70)	31 (16.80)	<.001
Physical examination, No. (%)			
Other rash (not EM)	71 (6.70)	12 (6.50)	.932
Joint swelling	50 (4.70)	22 (12.00)	<.001
Tender points	38 (3.60)	0 (0.00)	.004
Sensory abnormality	26 (2.50)	1 (0.50)	.164
Motor weakness	19 (1.80)	0 (0.00)	.096
Reported history of coinfection	133 (12.50)	6 (3.20)	<.001
EIA,^b^ No. (%)			<.001
Positive	342 (39.4)	121 (87.1)	
Negative	479 (55.2)	14 (10.1)	
Equivocal	46 (5.3)	4 (2.9)	
Immunoblot,^c^ No. (%)			<.001
-IgM -IgG	244 (34.7)	10 (6.9)	
-IgM +IgG	70 (10)	29 (20)	
+IgM -IgG	314 (44.7)	48 (33.1)	
+IgM +IgG	75 (10.7)	58 (40)	
Anti-infective medication: duration			.009
None, No. (%)	149 (14.00)	12 (6.50)	
1–30 d, No. (%)	316 (29.80)	88 (47.80)	
31–90 d, No. (%)	342 (32.20)	65 (35.30)	
91–182 d, No. (%)	121 (11.40)	15 (8.20)	
>183 d, No. (%)	120 (11.30)	3 (1.60)	
Unknown, No. (%)	13 (1.20)	1 (0.50)	
Median (p25–p75)	42 (21–90)	30 (21–51)	
Mean (range)	90 (0–2555)	43 (0–240)	
Total No. of anti-infective medications (%)			<.001
0	149 (14.00)	12 (6.50)	
1	511 (48.20)	103 (56.00)	
2	202 (19.00)	48 (26.10)	
3	93 (8.80)	17 (9.20)	
4	50 (4.70)	1 (0.50)	
5 or more	56 (5.30)	3 (1.60)	
Anti-infective medications, No. (%)			
Doxycycline	743 (70.00)	157 (85.30)	<.001
Ceftriaxone	203 (19.10)	33 (17.90)	.702
Amoxicillin	135 (12.70)	34 (18.50)	.035
Cefuroxime	94 (8.90)	13 (7.10)	.423
Azithromycin	101 (9.50)	3 (1.60)	<.001
Total No. of tests per individual (%)			<.001
None	22 (2.10)	10 (5.40)	
1 or 2	449 (42.30)	118 (64.10)	
3 or more	590 (55.60)	56 (30.40)	

Abbreviations: EIA, enzyme immunoassay; EM, erythema migrans; LD, Lyme disease.

^a^Patients with possible LD were not included for this comparison.

^b^Sample size for EIA is 1006 due to missing data.

^c^Sample size for immunoblots is 848 due to missing data.

### Predictors for Patients Without Current Lyme Disease

Multivariable logistic regression analysis (Table 3) yielded the following independent predictors for patients without current Lyme disease (n = 1061): female gender (odds ratio [OR], 1.56; 95% confidence interval [CI], 1.08–2.25), symptom duration >3 months (OR, 8.78; 95% CI, 5.87–13.1), higher number of symptoms (OR per additional symptom, 1.08; 95% CI, 1.02–1.13), greater number of Lyme disease laboratory tests (OR per additional laboratory test, 1.17; 95% CI, 1.03–1.32), and reported history of coinfections (OR, 3.13; 95% CI, 1.14–8.57). When further examined, symptom duration showed a direct correlation with the likelihood of patients not having current Lyme disease in the multivariable model. Our model estimated that in those with symptom duration from 3 to 6 months, the likelihood of not having current Lyme disease was 2.3 times higher than for similar patients with a symptom duration of 0–3 months (95% CI, 1.4–4), and the likelihood of not having current Lyme disease was 6.0 times higher when comparing those with a symptom duration >6 months to the 3–6-month group (95% CI, 3.6–10.0). Only joint swelling was associated with active/recent Lyme disease (OR without Lyme disease, 0.38; 95% CI, 0.19–0.874). Although univariate analysis demonstrated that an antimicrobial treatment duration of more than 3 months was associated with patients without current Lyme disease, it was not statistically significant in the multivariable model (*P* = .56).

**Table 3.** Logistic Regression Models: Likelihood Patients Do not Have Active/Recent LD as Explanation for Symptoms^a^

	Univariate Analysis		Multivariate Analysis^b^		
Covariates	OR (95% CI)	*P* Value	OR (95% CI)	*P* Value
Age (per 5 additional y)				
Age <40 y	1.225 (1.073–1.399)	.003	1.1 (0.935–1.286)	.255
Age 40–65 y	0.876 (0.779–0.986)	.029	0.932 (0.816–1.065)	.302
Age >65 y	1.202 (0.850–1.700)	.299	1.206 (0.861–1.691)	.276
Gender				
Female	1.945 (1.419–2.666)	<.001	1.561 (1.084–2.246)	.017
Male (reference)	1.0		1.0	
Symptom duration >3 mo	11.43 (7.92–16.49)	<.001	8.78 (5.87–13.13)	<.001
Symptom duration ≤3 mo (reference)	1.0		1.0	
Symptom count (per additional symptom)	1.147 (1.099–1.197)	<.001	1.076 (1.024–1.130)	.004
No. of lab tests (per additional lab)	1.367 (1.216–1.538)	<.001	1.167 (1.033–1.318)	.013
Treatment duration ≥3 mo	2.828 (1.742–4.593)	<.001	1.172 (0.692–1.985)	.555
Treatment duration <3 mo (reference)	1.0		1.0	
History of other infection	4.252 (1.847–9.789)	.001	3.129 (1.142–8.569)	.027
No history of other infection (reference)	1.0		1.0	
Joint swelling	0.364 (0.215–0.618)	<.001	0.378 (0.192–0.741)	.005
No joint swelling (reference)	1.0		1.0	

Abbreviations: CI, confidence interval; LD, Lyme disease; OR, odds ratio.

^a^Patients with possible LD were not included in these models.

^b^Sample size for the multivariate analysis is 1231, due to missing data.

Of the 911 patients without current Lyme disease, alternative diagnoses were made by the infectious diseases clinicians for 607 (66.6%) patients (a more detailed analysis will be reported separately).

## DISCUSSION

In this large population referred for presumptive Lyme disease, 1061 (84.1%) were judged not to have active/recent Lyme disease, yet 86% had received at least 1 course of antimicrobials to treat Lyme disease, and 37% had received 2 or more courses of anti-infectives. Fatigue and arthralgia were the most frequent complaints in all groups, but patients without current Lyme disease had lower rates of objective findings such as joint swelling or a positive 2-tier serologic test, and they tended to be female. Patients without current Lyme disease also had more symptoms and more laboratory tests performed to diagnose Lyme disease. Though it is the most common vector-borne illness in the United States, the high rates of Lyme disease misdiagnosis and overtreatment in this referred population may suggest that the actual incidence of infection is less than what has been calculated when using information based on insurance claims data [14–16].

Recent studies have highlighted that in the US adult population, 20.4% suffer from chronic pain, 10.2%–15.7% have fatigue, and 11.2% of persons ≥45 years old describe subjective cognitive dysfunction [17, 18]. Given the frequent lack of an identified cause for these common symptoms, Lyme disease appears to be an attractive diagnosis for otherwise unexplained, long-standing problems that are termed by some “chronic Lyme disease” [7–11, 19–22]. In this study population, these were the leading complaints, and the longer the duration of symptoms, the less likely patients were to have authentic Lyme disease. Among patients who had symptoms for more than 3 months, the likelihood of Lyme disease being the correct diagnosis was 8.8 times less frequent than among those with a shorter duration of symptoms.

The scientifically counterintuitive notion that an infection requiring antibiotics is the cause of long-term clinical symptoms highlights a fundamental problem centering on accurate diagnoses of Lyme disease. Studies conducted between 1990 and 2002 found Lyme disease diagnosis rates of 33.0%–49.5% in referral populations, whereas a 2012 study from the United Kingdom and the current study demonstrated fewer accurate Lyme disease diagnoses, with rates of 23.0% and 27.8%, respectively (Table 4). Some of these earlier studies were performed when first-generation Lyme disease serologic assays yielded higher false-positive rates than the current 2-tier standardized testing adopted in 1994–1995 [12]. Although one would anticipate improved diagnostic accuracy with improved specificity since the introduction of standardized 2-tier serology, the findings of this study suggest the opposite, with results that fall into a similar range of 9.6%–15.0%, as cited in recent French referral populations [23–25].

**Table 4.** Previous Reports of Patients Referred for Lyme Disease in Endemic Regions

Author [Ref. #]	Year	Location	Total No. of Patients	Age, y	Male, %	Current Lyme Diagnosis, No. (%)	Total Lyme Diagnosis (Current + Remote), No. (%)
Sigal [10]	1990	NJ	100	35.1 (median)	32	N/A	37 (37)
Steere [19]	1993	MA	788	38^a^	56^a^	180 (23)	336 (42.6)
Rose [20]	1994	PA	227	N/A^b^	N/A	N/A	75 (33.0)
Feder [11]	1995	CT	146	9.9 (mean)	53	N/A	87 (59.6)
Reid [9]	1998	CT	209	40^c^	48^c^	44 (21)	84 (40.2)
Qureshi [8]	2002	NY	216	N/A^d^	60	68 (31.5)	107 (49.5)
Cottle [39]	2012	UK	115	42 (median)	44	N/A	27 (23)
Jacquet [23]	2018	France	468	51.4 (mean)	50	69 (15)	N/A
Haddad [24]	2018	France	301	50 (median)	61	29 (9.6)	N/A
Bouiller [25]	2018	France	355	N/A	N/A	48 (13.5)	N/A
Current study	2019	MD	1261	45.7 (mean)	38.2	184 (14.6)	350 (27.8)^e^

^a^Mean age and male gender in those with current LD.

^b^Aged 1–19 years.

^c^Median and male gender in those with current LD.

^d^Age <19 years.

^e^Includes possible Lyme disease.

How to explain these strikingly high rates of misdiagnosis in recent years cannot be directly determined from the study data. However, the characteristics of those with remote Lyme disease were similar in this study to the characteristics of those without Lyme disease, suggesting that regardless of test results, Lyme disease is the diagnosis used to explain the mostly subjective symptoms.

A possible explanation for high rates of misdiagnosis is that the reporting of Lyme disease serologic testing is prone to misinterpretation. Problems range from attributing the presence of even a single band on an immunoblot as representing active infection to diagnosing Lyme disease in patients with >30 days of symptoms based on a positive IgM immunoblot without a positive IgG immunoblot, both contrary to current recommendations [26, 27]. This study does confirm that misinterpretation of a positive IgM immunoblot was associated with the incorrect use of antibiotic treatment, similar to prior studies [26, 28]. Additionally, 40%–60% of patients with a remote history of Lyme disease can have a persistently positive IgG and/or IgM serology even a decade or more after successful treatment, leading to the mistaken conclusion that Lyme disease is an explanation for unconnected symptoms [29].

Although improved laboratory reporting methods would help lessen confusion, given more than 2 decades of familiarity with 2-tier testing, it is unlikely that more provider education will substantially help, leaving next-generation Lyme disease testing a possible path toward more accurate diagnoses [30]. However, improved testing may not change the perspective of health care providers who diagnose chronic Lyme disease in patients with unexplained chronic or unexplained symptoms, regardless of negative laboratory test results for Lyme disease [31–33].

Four or more weeks of antibiotics were received by 53.4% in the entire study sample regardless of Lyme disease status. Both patients and clinicians may look to antibiotics for speeding the resolution of persistent symptoms despite evidence to the contrary [5]. Inappropriate use of longer-term treatment with antibiotics may lead to substantive delays in reaching proper diagnoses and/or may lead to adverse reactions such as alterations in the human microbiome, *Clostridioides difficile* colitis, central venous catheter–associated infections, venous thromboses, severe allergic reactions, and even fatalities [9, 34, 35].

Although Lyme disease is overdiagnosed in this population, a novel finding not previously reported in a large series is the frequency of diagnosis of coinfections in addition to Lyme disease (both *Ixodes* and non-*Ixodes* tick-borne, plus non-tick-borne infections), which were believed to be contributing to the symptoms for 11% of study patients. Babesia, Epstein-Barr virus, *Bartonella* and *Ehrlichia* spp. infections were the most frequent infections codiagnosed before referral. These patients appeared to receive multiple antibiotics at their initial diagnosis of Lyme disease directed to coinfections, or when no improvement was noted, coinfection diagnoses were introduced and treatments were prescribed. Although *Ixodes* spp. ticks may transmit human pathogens other than *B. burgdorferi*, such as *Anaplasma phagocytophilum, Babesia microti,* and deer tick virus, authenticated coinfection is seen mainly during acute illnesses rather than in people with long-term complaints [36]. A systematic review revealed no support for the hypothesis that atypical tick-borne coinfections are responsible for persistent symptoms in patients with chronic, nonspecific illnesses [37, 38]. Patients with longstanding complaints who are told they have multiple tick-borne or non-tick-borne infections in addition to Lyme disease should provide a strong signal to clinicians that these infectious etiologies are likely to be based on nonvalidated principles and are not an explanation for the patient’s symptoms.

### Limitations

This study has limitations as a retrospective, observational study subject to missing data, referral bias, clinical judgments, and unmeasured confounders. As a single-center study at an academic hospital with medically complicated patients, this population may differ from community or other hospital settings. Also, the available clinical data did not allow a precise determination of whether patients were referred by providers or were self-referred. However, as the majority of patients were treated with antibiotics for Lyme disease, it is likely that Lyme disease was a diagnosis made by a health care professional.

The study design did not allow for investigation as to how many patients with Lyme disease were previously coded for billing purposes or reported to state health departments, which might give further insight into the accuracy of such data. Any self-reported history of coinfections and treatment thereof were not evaluated for validity if medical record data were missing. Also, judgments made by infectious diseases clinicians in this study group could have influenced results, as collected retrospective data were heterogeneous by nature. Lastly, the decision to include remote Lyme disease patients with those without Lyme disease may represent different group effects, although characteristics appeared similar when analyzed separately.

## CONCLUSIONS

Our evaluation of patients seen for Lyme disease in infectious diseases consultation found that the majority had neither Lyme disease nor a convincing history of prior Lyme disease. One-half of the referred patients received antibiotics for longer durations than recommended regardless of their diagnosis. This 13-year study suggests that patients and clinicians may be influenced by alternative, non-evidence-based medical practices, or could be confused by nonvalidated laboratory test results or interpretations. Among these referred patients, female gender, symptoms for longer than 3 months, a higher number of subjective symptoms, having had multiple Lyme disease tests, and a reported history of other concurrent infectious diagnoses, including tick-borne coinfections, are all unlikely to suggest Lyme disease as an explanation for the presenting symptoms.

## Supplementary Data

Supplementary materials are available at *Open Forum Infectious Diseases* online. Consisting of data provided by the authors to benefit the reader, the posted materials are not copyedited and are the sole responsibility of the authors, so questions or comments should be addressed to the corresponding author.
